# Time Modulated Arrays: From their Origin to Their Utilization in Wireless Communication Systems

**DOI:** 10.3390/s17030590

**Published:** 2017-03-14

**Authors:** Roberto Maneiro-Catoira, Julio Brégains, José A. García-Naya, Luis Castedo

**Affiliations:** Department of the Electronics and Systems, University of A Coruña, Facultad de Informática, Campus de Elviña s/n, A Coruña 15071, Spain; roberto.maneiro@udc.es (R.M.-C.); julio.bregains@udc.es (J.B.); luis@udc.es (L.C.)

**Keywords:** antenna arrays, digital communications, time modulated arrays

## Abstract

Time-modulated arrays (TMAs) are electromagnetic systems whose radiated power pattern is controlled by the application of variable-width periodical pulses to the individual elements. The nonlinear nature of the array operation causes the appearance of radiation patterns at the harmonic frequencies of such periodic pulses. The technique can be used for improving the side-lobe level (SLL) topology of the radiation pattern at the central frequency and/or to profitably exploit the harmonic patterns in order to supply smart antenna capabilities. Among the latter features, the TMA harmonic beamforming takes on special importance due to its attractive trade-off performance-hardware complexity. From this perspective, TMAs are sensors capable of transforming the spatial diversity of a communication channel into frequency diversity, thus improving the performance of a wireless communication. In addition to a walk through the origins of the concept, and a brief analysis of the mathematical fundamentals, this paper organizes the prolific state of the art of TMAs in two major thematic blocks: (1) TMA design from an antenna perspective; and (2) TMA design from a signal processing perspective.

## 1. Introduction

Wireless services demanding more mobility, capacity, and robustness have placed wireless communications as the fastest growing sector of the telecommunication industry nowadays. As a result, the increasingly limited wireless medium has to be smartly exploited and antennas are called to play an important role to achieve such a goal. A proper combination of antenna features and signal processing allows us to jointly and efficiently act in the space, frequency, and time domains in order to provide suitable alternatives to the complex task of faithfully receiving the desired signals and efficiently mitigating the unwanted ones.

One of the primary objectives of an intelligent system supported by a sensor network is its sustainability. The investigation of new technologies allowing for reducing the power consumption of sensor networks is aimed towards the design of smart antennas with low loss, muting and adaptive beamforming features. Additionally, such a smart antenna technology has to consider the hardware complexity, making the solution feasible in the context of sensors. Moreover, antenna devices pertaining to sensor networks must be capable of sensing the external electromagnetic environment, thus properly reconfiguring the radiation characteristics of the generated field to guarantee both the required quality of service of the communication link and a reduced power consumption.

However, some technical challenges remain in order to develop the adequate antenna technologies capable of supporting the aforementioned features in a limited physical space that the mobility demand dictates. In fact, today’s wireless standards consider multiple-antenna techniques [1,2,3] in order to exploit space diversity, spatial multiplexing and beamforming to achieve better levels of reliability and capacity. Such advantages, however, are obtained at the expense of increased system complexity, which may be unaffordable in small-size and low-cost sensor devices with a required low-power consumption.

The concept of TMA is a feasible multi-antenna technique that provides a significant hardware simplification. Furthermore, TMA, typified as unconventional phased arrays architectures, have been intensively researched during the last few years at an increasing pace [4].

### 1.1. What is a TMA?

**Figure 1:**
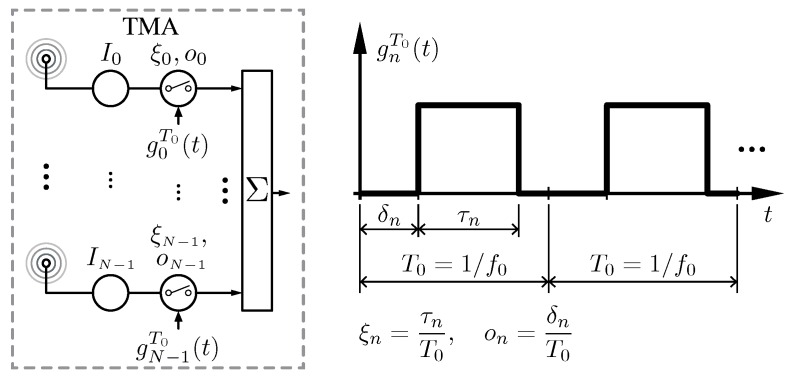
On the left hand side, the block diagram of a linear time-modulated array (TMA) with *N* elements implemented with radio-frequency (RF) switches, with $I_{n}$ being the static excitation of the *n*-th element of the array. On the right hand side, the periodic ($T_{0}$) pulse train that governs the *n*-th switch, $g_{n}^{T_{0}}\left(t\right)$, which is characterized by the normalized pulse duration $\xi_{n}$ and the normalized switch-on instant $o_{n}$.

TMA, in their simplest configuration, are antenna arrays whose radiated power patterns are controlled by periodically enabling and disabling the excitations of the individual array elements, as illustrated in Figure 1. Hardware implementations of TMA simplify considerably since they can be done with switches rather than the complex weight multiplications of conventional arrays.

The TMA radiated field with *N* isotropic elements distributed along the *z*-axis is given by [5]

(1) $$\begin{array}{c} F\left(\theta ,t\right)=e^{j2\pi f_{c}t}\sum_{n=0}^{N-1}g_{n}^{T_{0}}\left(t\right)I_{n}e^{jkz_{n}\cos\theta }\in C, \end{array}$$

where $g_{n}^{T_{0}}\left(t\right)$ is a periodic function with a fundamental period $T_{0}=1/f_{0}$, which governs the *n*-th array element, with n=0,⋯,N−1; $I_{n}=\left|I_{n}\right|e^{j\varphi_{n}}$ and $z_{n}$ represent the *n*-th antenna element complex static current excitation in its polar form ($\left|I_{n}\right|$ is its modulus and $\varphi_{n}$ is its phase) and the position on the *z*-axis, respectively; $f_{c}$ is the carrier frequency of the incoming signal; *θ* is the angle with respect to the array main axis; and k=2π/λ is the wave number for a wavelength $\lambda =c/f_{c}$.

Since $g_{n}^{T_{0}}$ is a periodic signal, it can be represented by the following exponential Fourier series:

(2) $$\begin{array}{c} g_{n}^{T_{0}}\left(t\right)=\sum_{q=-\infty }^{\infty }G_{nq}e^{jq2\pi f_{0}t}\in R, \end{array}$$

where, in a conventional TMA, $g_{n}^{T_{0}}\left(t\right)$ is a periodic rectangular pulse train with duration $\tau_{n}$ and delay $\delta_{n}$ (see Figure 1). In such a case, $G_{nq}$ is given by [6]

(3) $$G_{nq}=\xi_{n}\mathrm{sinc}\left(q\pi \xi_{n}\right)e^{-jq\pi (\xi_{n}+2o_{n})}\in C,$$

where sinc(x)=sin(x)/x denotes the sinc function, $\xi_{n}=\tau_{n}/T_{0}\in \left(0,1\right)\subset R$ are the normalized pulse durations, and $o_{n}=\delta_{n}/T_{0}\in \left[0,1-\xi_{n}\right]\subset R$ are the normalized pulse delays. In some cases, and according to the properties of the pulses, $g_{n}^{T_{0}}\left(t\right)$, the dependency on $o_{n}$ can be avoided, yielding $G_{nq}=\xi_{n}\mathrm{sinc}\left(q\pi \xi_{n}\right)e^{-jq\pi \xi_{n}}$, but, in this work, we consider the general form. Note that for q=0 we have $G_{n0}=\xi_{n}$. Substituting Equation (2) into Equation (1) allows for rewriting the TMA radiated field as

(4) $$\begin{array}{cc} F(\theta ,t) & =\underset{q=0}{\underset{︸}{\left[\sum_{n=0}^{N-1}I_{n}\xi_{n}e^{jkz_{n}\cos\theta }\right]e^{j2\pi f_{c}t}}}+\\ & +\left[\sum_{n=0}^{N-1}\sum_{\begin{array}{c} q=-\infty \\ q\neq 0 \end{array}}^{\infty }I_{n}\underset{G_{nq},\;q\neq 0}{\underset{︸}{\xi_{n}\mathrm{sinc}\left(q\pi \xi_{n}\right)e^{-jq\pi (\xi_{n}+2o_{n})}}}e^{jkz_{n}\cos\theta }\right]e^{j2\pi (f_{c}+qf_{0})t}. \end{array}$$

Observe that the first term in Equation (4) is the array factor at the fundamental mode (q=0) frequency $f_{c}$, which shows that, by means of the normalized pulse durations $\xi_{n}$, we are able to reconfigure exclusively the magnitude of the excitations and thus control the radiation pattern. On the other hand, the second term in Equation (4) reveals the existence of sideband radiation (SR) at frequencies $f_{c}+qf_{0}$ (harmonics). Note that by modifying $\xi_{n}$ and/or $o_{n}$ we can also control the radiation pattern at each sideband harmonic, in this case being able to simultaneously reconfigure the magnitude and the phase of the excitations. The SR can be either minimized or, as explained in the forthcoming sections, profitably exploited.

### 1.2. TMA Features

As shown above, the periodic modulation of a TMA is a nonlinear operation that generates sideband signals radiated at frequencies shifted from the carrier frequency at multiples of the time-modulation rate $f_{0}$. The time parameters which govern the TMA (specifically, the switch-on durations $\tau_{n}$ and the switch-on delays $\delta_{n}$ [7,8]) can be optimized to:

1. exploit the fundamental pattern only —at the carrier frequency $f_{c}$— with the aim of achieving ultra-low side-lobe level (SLL) while minimizing the SR, or
2. profitably exploit the harmonic patterns, endowing the TMA with smart antenna capabilities.

Hence, and according to the second point, by adapting the harmonic patterns to the wireless channel, and by combining its corresponding outputs, TMA are able to perform adaptive beamforming with the benefit of using a single RF front-end. In this regard, the fact of avoiding the utilization of as many RF chains as the number of multipath components to be exploited, offers a series of key advantages:

- Hardware simplicity, with the subsequent impact on the size and the cost of the system.
- Power consumption, as it has been remarked and properly quantified in recent papers like (Table I, [2]) and (Tables I and II, [3]), which have also proposed hybrid analog–digital architectures for beamforming. Indeed, in a fully digital implementation of an linear beamforming network (LBFN), the number of required RF chains, *L*, must be equal to the number of antenna elements *N*. In practice, however, a relationship N≫L is often preferable due to a number of reasons, the power consumption per RF being the front-end one of the most important ones.
- Nonexistence of issues related to synchronization, phase coherence or coupling between different RF chains.

In summary, the application of periodic pulses at the antenna level has the effect of converting space diversity into frequency diversity and, thus, a single RF front-end is enough to exploit such a space diversity.

## 2. State of the Art

### 2.1. The Origin of the TMA Concept

The pioneering idea of using the time as an additional degree of freedom to control the radiation pattern of an antenna was proposed by Shanks and Bickmore, from the Microwave Laboratory at the Hughes Aircraft Company, Glendale, CA, USA in 1959 [9]. The authors introduced theoretically this entirely new idea in antenna design and suggested several possible physical antenna configurations, such as a square wave switched linear array. However, they employed a common parabolic dish antenna in their first experimental tests and applied a nutation movement to the primary horn with a frequency of 133 Hz. With this technique, they achieved a multi-pattern operation obtaining simultaneously a sum diagram at the fundamental frequency and a difference diagram at the first sideband harmonic.

In 1963, Kummer et al. —also from Hughes Aircraft Company— experimentally extended the concept of “time-modulated antenna” to antenna arrays [5], thus coining the term TMA. They obtained ultra-low side lobe patterns by inserting a set of RF switches —programmed according to a predetermined periodic time sequence— in the feed network of an 8-element waveguide slotted linear array working at the X-band. Kummer et al. stated that the higher the SLL improvement by applying time modulation, the higher the cost in the system gain. The source of this gain cost is the nonlinear nature of the periodic modulation, which inherently generates sideband radiated loss signals. Consequently, SR had been primarily identified as a phenomenon that severely degrades the TMA gain. As a matter of fact, Kummer et al. focused on two representative examples. On the one hand, they selected an initial static Dolph–Tschebyshev pattern with −30 dB SLL improved down to about −40 dB —by applying time modulation implemented by means of RF switches —at the expense of a modest loss in the antenna gain (0.3 dB). On the other hand, they considered a uniform static aperture distribution —with an SLL of −13 dB— improved down to −40 dB through time modulation with a much higher price to be paid (2.4 dB) in terms of the antenna gain. In this way, Kummer et al. have implicitly opened the door to future research that would be focused on minimizing the SR while improving the SLL through the application of the TMA technique.

### 2.2. TMA Design under an (Exclusive) Antenna Perspective

Most of the research works involving TMA have been developed under an antenna designer point of view. More specifically, they focus on the synthesis of an appropriate shape of the radiation pattern. We therefore classify such works depending on the patterns that are synthesized, i.e., fundamental or harmonics.

#### 2.2.1. Fundamental Mode Pattern

Although it represents a natural and immediate area worth of investigation, the development of methods to minimize the SR did not start until the early 2000s when the first optimization algorithms emerged. The reason is that the sophisticated methods necessary to carry out this kind of optimization arrived simultaneously with the development of more powerful computers. In this sense, Yang et al. introduced in 2002 the TMA synthesis supported by systematic algorithms, capable of optimizing the SLL and the SR simultaneously by using differential evolution algorithms [10]. In subsequent works, and with the same optimization objectives, other algorithms were considered: genetic algorithms [11], simulating annealing [12,13], particle swarm optimization [14], and artificial bee colony [15]. Further works involved additional parameters in the multi-objective optimization, like the half power beam width [16]. Other innovative —and structurally different— techniques have been recently proposed to minimize the SR based on modulating the RF switches with non-uniform [17] or multiple [18] time modulation periods.

Later on, an additional degree of freedom was introduced in the synthesis process by considering the switch-on instants of the periodic rectangular pulses —in addition to the pulse durations— applied to the antenna excitations [7,19]. In [20], the time modulation period was split into several time steps with variable lengths and, for each time step, the switch-on and switch-off times were optimized via the differential evolution algorithm to improve the SR radiation. In a further development, the impact of the periodic pulses time shift on the SR was theoretically analyzed [8]. Likewise, hardware solutions based on sub-arraying techniques [21] or the use of fixed bandwidth elements [22], as well as the impact of pulse shaping [23], were also investigated to reduce the SR.

In addition to linear geometrical configurations, planar and even conformal TMA were also analyzed and optimized in terms of SLL and SR [19,24,25,26], as well as planar adaptive absorbers for radar based on phase-switched screen, whose design was modeled by means of the time-switched array theory [26].

Another key aspect in the TMA technique is the antenna gain and how it is affected by both the SR and the switching network. In [27], Yang et al. characterized the TMA gain and quantified the TMA efficiency. More specifically, they separated the switching network efficiency (which accounts for the time fraction that the switches are off) and the TMA power efficiency (the ratio between the fundamental mode mean radiated power and the total mean radiated power, i.e., the useful radiated power divided by total radiated power) being the total efficiency of the TMA the product of both. In order to improve the switching efficiency. In [28], the utilization of single-pole double-throw (SPDT) switches was proposed in such a way that a single RF switch governs two adjacent elements of the array, so that, whenever complementary time sequences are synthesized, the off time is reduced to zero, and consequently the switching efficiency is set to an almost ideal value. More sophisticated structures based on the use of a reconfigurable power divider/combiner have been evaluated in [29] in order to improve the switching network efficiency of the array.

On the other hand, since the fluctuations of the antenna gain —due to the application of a periodic on–off modulation to the static excitations— may severely affect the behavior of a received signal, some research works focused on the switching sequence, primarily to keep the instantaneous directivity of the main beam constant [30,31]. TMA were also analyzed —exclusively from the radiation properties point of view— so as to be exploited in different applications, e.g., for the suppression of interferences and undesired signals impinging on the antenna aperture. Such a capability of adaptive nulling in time-varying scenarios was evaluated in [32] by Poli et al., and completed in [33]. Further examples are the synthesis of the so-called power-patterns for wide coverage purposes [34,35], or radar applications [36,37].

Apart from all of these applications, tools for a more accurate analysis of TMA were also developed. In this sense, and in order to evaluate rigorously some nonlinear performance aspects of TMA as a radiating system (e.g., the effects of mutual coupling between elements), a full-wave computation of the radiated far field was addressed by Masotti et al. in [38] by properly developing a multi-domain computer aided design platform. Aligned with this research topic, a full-wave analysis of the instantaneous and average behaviors of TMA is available in [39].

#### 2.2.2. Harmonic Patterns

As already pointed out, the SR is not necessarily a damaging phenomenon and can be profitably exploited to improve the performance of a TMA. As a matter of fact, the exploitation of the harmonic patterns endows TMA with smart-antenna capabilities. In this sense, the seminal work by Shanks [40] theoretically introduced the beam-scanning capabilities of TMA and, as an example, such features have been efficiently applied to an experimental prototype in [41].

Adaptive beamforming was originally studied from a practical and simplified point of view by Li et al. [42], focusing on the harmonic patterns synthesis in [6], and even taking advantage of time redundancy in [43]. Another strategy for beamforming through pulse splitting techniques was investigated in [44]. In such a technique, based on particle swarm optimization, two patterns were simultaneously generated: the first one at the central frequency and the second one at a preselected harmonic with an arbitrary order, keeping the SR below a given level. An example of how harmonic beamforming with TMA can be profitably applied, in this case to blanking in spectrometry and radar, was proposed in [45].

Beam steering with TMA was also introduced by Li et al. [46], and a more elaborated version, with control of the side-lobe level, was addressed in [47]. Applications of beam steering like direction finding or direction of arrival (DOA) estimation were analyzed in [48,49,50].

Reflector arrays were also considered for being implemented using time modulation. Wang et al. introduced the concept of time-modulated reflector array (TMRA) in [51], which was analyzed more deeply in [52]. A TMRA makes use of the topology of a conventional reflector array, i.e., consists of a matrix of scattering elements which are illuminated by a feed horn but, instead of phase shifters, it uses discrete time switching to achieve beam steering or beamforming functions. As a result, the radiation pattern is controlled by adjusting the time-domain scattering. It was also proven that, by employing double-layer designs [53], it is possible to increase the energy efficiency of TMRA.

Another interesting field for TMA applications is wireless power transmission, introduced by Masotti et al. in [54]. In this case, the real-time beamforming features of TMA are properly exploited following a two-step procedure: firstly, to precisely localize the tag to be powered and, subsequently, to perform the directive wireless power transmission.

In recent investigations, the TMA philosophy was involved in more complex hybrid solutions contributing to improve the performance of the overall antenna system. In this way, time modulation was applied —together with in-phase and quadrature (I/Q) modulation— to phased arrays in order to generate a scanning beam at the single positive sideband [55]. Time modulation was considered, jointly with *retrodirective* techniques, to synthesize *coaperture* antenna arrays [56] and also to obtain time-invariant spatial-fine-focusing beampatterns in frequency diverse arrays [57].

### 2.3. TMA Design under a Signal Processing Perspective

This section is divided into two parts. First, the initial approaches that have jointly considered TMA and wireless communications are introduced. Second, the models involving TMA together with linear digital transmitter and receivers are addressed. Multipath fading wireless channels together with diversity exploitation at the receiver are also considered. Finally, the efficiency of the TMA as well as how to improve it are taken into account, leading to the so-called enhanced time-modulated array (ETMA).

#### 2.3.1. Interaction of TMAs with Information Signals: First Approaches

Most of the above-referenced investigations exclusively focus on the synthesis of the antenna patterns. The fundamental and/or the harmonic components are manipulated in some way, without considering the interaction between the TMA technique and the signals sent or received by such an antenna. This subsection is devoted to those works that apply TMA to wireless communications.

As a starting point, it is necessary to find the signal frequency restrictions, i.e., the relationship between the time modulation frequency, the signal bandwidth, and the carrier frequency that must be satisfied in order to keep the integrity of the signals transmitted/received over the TMA. Those restrictions were initially stated by Shanks and Bickmore [9], and completed by Brégains et al. [58], where additional closed-form expressions for the total mean SR power and for the TMA power efficiency were properly derived.

The transmission of narrowband amplitude-modulation (AM) and frequency-modulation (FM) signals was theoretically analyzed by Li et al. [59] and, for the case of AM, experimentally proved in [60]. Other specific cases were also investigated like a dual-channel AM receiver [61], the transmission of linearly frequency modulated (LFM) signals [62], or the feasibility of implementing a dual function of radar and communication [63,64].

The TMA technology was also proposed—originally by Zhu et al.—to transmit direction-dependent signals in the context of secure communications [65,66,67]. The idea is based on generating a null level of SR in some desired directions and a high level of SR on other directions in order to distort the signal. Conditions of frequency overlapping between the signal replicas placed in adjacent harmonic frequencies must be satisfied, paradoxically contrary to those ones established in [9].

A first study on the signal-to-noise ratio (SNR) in a system with a receiver TMA was presented in [68] by Zhu et al. This work describes a specific case exploiting the fundamental pattern and the first positive harmonic to receive two different binary phase shift-keying (BPSK) signals over additive white Gaussian noise (AWGN) channels. The multibeam characteristics of TMA were also proposed for spatial multiplexing by He et al. [69], although the performance with broadband signals was not analyzed. More recent works focus on the hardware design of TMA applied to spatial filtering of signals [70], and even for interference suppression in mm-Wave communications [71].

Note that, although the TMA topic has been mainly considered from a theoretical point of view, several prototypes have already been described in the literature [41,42,46,49,50,56,60,69,70].

#### 2.3.2. Towards Using TMAs in Wireless Communication Systems

**Figure 2:**
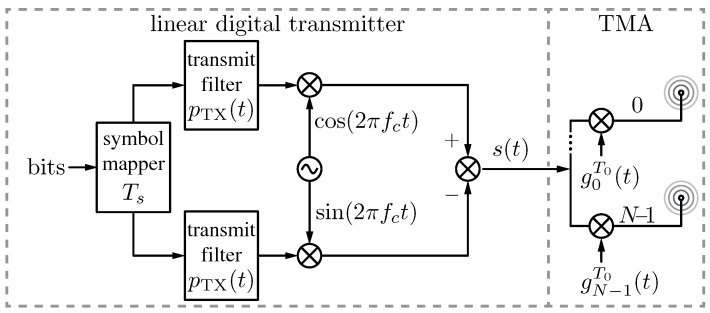
Transmitter block diagram considering a linearly modulated digital signal radiated through a time-modulated array (TMA) exploiting its fundamental pattern: $p_{\mathrm{TX}}\left(t\right)$ is the transmit filter, $f_{c}$ is the carrier frequency, s(t) is the TMA input signal, and $g_{0}^{T_{0}}\left(t\right),\cdots ,g_{N-1}^{T_{0}}\left(t\right)$ are the periodic ($T_{0}$) functions governing the *N* array elements.

None of the above-referenced works analyze in depth, and in a theoretical way, the influence of TMA on digital communication wireless systems. In this sense, the feasibility of TMA for digital communications has been investigated in [72]. This first insight towards the study of the impact of the TMA technique on the transmission of digital signals has focused on a particular family: linear modulations (Chapter 5, [73]). The main reason for this choice is that these signals exhibit the simplest schemes to be considered in any analysis pertaining to digital signal transmission systems. On the other hand, a TMA by itself imposes a nonlinear transformation—derived from the application of periodic pulses to the antenna excitations, as mentioned above—and, therefore, linear modulations avoid the introduction of additional nonlinearities in the communication system. The block diagram in Figure 2 illustrates the continuous-time version of the system under study, in which a linearly modulated digital signal s(t) is first generated by means of an in-phase and quadrature modulator at carrier $f_{c}$, and, next, s(t) is radiated through a TMA that exploits its fundamental pattern assuming a fast decay of the harmonics. Note that we restrict ourselves to the continuous-time model (thus the digital-to-analog converter (DAC) is not included) and that the digital information symbols are generated at a symbol period $T_{s}$. Accordingly, the restrictions to safeguard the integrity of the signal and the radiated power (useful and SR) under those conditions have been mathematically analyzed and quantified, both in the time and frequency domains.

- The referred restrictions are the following:
  1. $f_{0}\ll f_{c}$, with $f_{0}=1/T_{0}$ being the TMA fundamental frequency and $f_{c}$ the carrier frequency of the TMA input signal s(t) (see Figure 2).
  2. $f_{0}>B_{s}$, where $B_{s}=\left(1+\rho \right)/T_{s}$ is the bandwidth of the TMA input signal s(t), $T_{s}$ is the symbol period and ρ∈[0,1] is the so-called roll-off factor when the transmit filter $p_{\mathrm{TX}}\left(t\right)$ corresponds to the well known raised cosine filter [74].
- The radiated power through the TMA is given by:
  (5) $$P_{\mathrm{rad}}=G_{\mathrm{TMA}}P_{ui}=\left(G_{{\mathrm{TMA}}_{0}}+G_{{\mathrm{TMA}}_{\mathrm{SR}}}\right)P_{ui},$$
  where $P_{ui}=4\pi D_{u}$ (expressed in watts) represents the power radiated by an isotropic antenna transmitting a linearly modulated equivalent baseband digital signal u(t)∈C with a power density $D_{u}$ (it is possible to consider $D_{u}=E_{s}\left(4-\rho \right)/4$, with $E_{s}$ being the average transmit symbol energy and *ρ* the roll-off factor of the raised cosine filter), whereas $G_{\mathrm{TMA}}$ represents the TMA power transfer function (dimensionless) accounting for both the array geometry and the time-modulated elements radiating a carrier signal. The $G_{\mathrm{TMA}}$ expression coincides with the array total power in (Equation (30), [58]) and is employed to separate the useful power for q=0 ($G_{{\mathrm{TMA}}_{0}}$) from the harmonic SR losses for q≠0 ($G_{{\mathrm{TMA}}_{\mathrm{SR}}}$).

**Figure 3:**
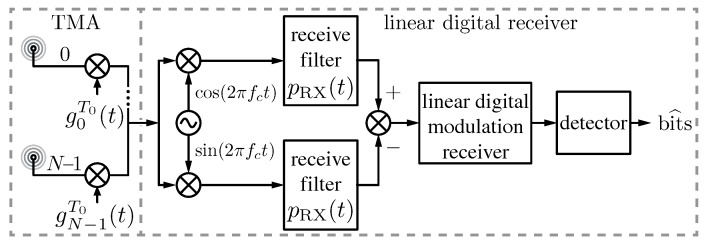
Receiver block diagram based on a linear digital modulation scheme incorporating a TMA. The notation is the same as in Figure 2 except $p_{\mathrm{RX}}\left(t\right)$, which denotes the receive filter.

Once the conditions for faithfully transmitting digital signals over a TMA have been stated, as well as the SR losses characterized, the next step consists in investigating the impact of a TMA on the performance of a digital communication system. Aligned with this idea, the performance —or quality— of the communication link in which the TMA is involved was studied in [75]. In such a work, the TMA exclusively exploits its fundamental pattern (a pencil beam pattern in this case). The block diagram of the corresponding digital communication system under study is shown in Figure 3. Such a study characterized, for the first time and from a theoretical point of view, the bit error rate (BER) (Chapter 6, p. 171, [73]) of a linearly modulated digital communication system with a TMA at the receiver for the simplest case of an AWGN channel model (Chapter 1, p. 33, [76]).

**Figure 4:**
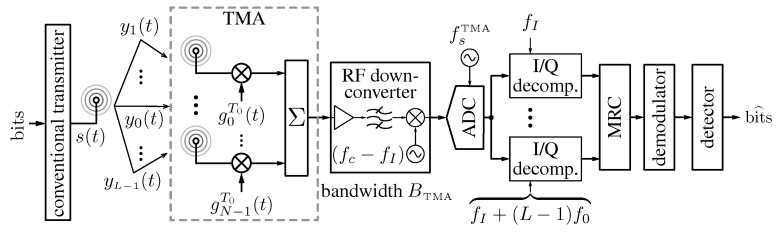
Block diagram of a system consisting of a conventional linear digital transmitter equipped with an omni-directional antenna and a receiver with a TMA designed for exploiting channel diversity at the receiver through maximum ratio combining (MRC).

However, the major potentialities of TMA for wireless communications are unavoidably related to the exploitation of their harmonic patterns. In this sense, most works available in the literature address the harmonic beamforming capability exhibited by TMA focusing exclusively on a radiation point of view. Accordingly, different scenarios of harmonic beamforming have been properly investigated, e.g., the conversion of spatial diversity into frequency diversity [6,42,43,44,45], the mitigation of interfering signals [6,42], the orthogonality between patterns [66,69], and the advantages of time redundancy [43]. However, all of those works have neglected a crucial aspect in the system performance when communications signals are involved: the TMA efficiency. Therefore, a more ambitious extension of the analysis in [75] had to be undertaken, by involving the TMA in a multipath fading communication where the antenna efficiency played a key role. Hence, the true challenge at this point is the synthesis of an efficient harmonic beamforming where it is not enough to satisfy certain conditions in terms of locations of maxima and nulls, but to safeguard the TMA efficiency and, consequently, the average SNR at the receiver (recall that the gain per beam path is proportional to the TMA efficiency [27]). The pioneering work involving the efficiency in the TMA beamforming design is [77], which is devoted to the performance analysis of TMA for the angle diversity reception of digital communication signals. Figure 4 shows the system block diagram whose performance is analyzed in [77]: a narrowband wireless communication system using a conventional *M*-ary linear digital modulation assuming an omnidirectional antenna at the transmitter, and a TMA, together with a maximum ratio combining (MRC) at the receiver, to exploit the channel diversity through the beamforming with the first L−1 positive TMA harmonic patterns.

According to Figure 4, once the transmitted signal s(t) arrives through a multipath channel at the receiver, we obtain *L* different replicas $y_{0}\left(t\right),\cdots ,y_{L-1}\left(t\right)$ constituting the TMA input. Each of the *L* replicas at the TMA output appears at a different (harmonic) frequency $f_{l}=f_{c}+l\cdot f_{0}$, with l=0,⋯,L−1. Assuming that $f_{0}$ has been correctly selected according to the bandwidth $B_{s}$ of s(t), the TMA output signal occupies a total bandwidth $B_{\mathrm{TMA}}\geq \sum_{l=0}^{L-1}B_{s}=L\cdot B_{s}$. After the RF down-conversion at a frequency $f_{c}-f_{I}$ (being $f_{I}$ the lowest intermediate frequency), the obtained signal is sampled at a frequency $f_{s}^{\mathrm{TMA}}>2B_{\mathrm{TMA}}$ (fulfilling the Nyquist criterion) and each of the resulting *L* digital signals is I/Q decomposed (including the corresponding receive filter) at a different intermediate frequency $f_{l_{IF}}=f_{I}+l\cdot f_{0}$, with l=0,⋯,L−1. Finally, the obtained *L* complex-valued signals are combined in an MRC and the resulting signal is demodulated to obtain the received bits. Notice that, for the sake of simplicity, channel estimation and equalization are assumed to be perfect. Note also that the non-linear operation carried out by the TMA allows for employing a single RF branch (with increasing bandwidth) followed by a single analog-to-digital converter (ADC) (with a higher sampling rate) to exploit *L* signal replicas impinging at the TMA. Consequently, the complexity of the receiver structure is simplified and, above all, its power consumption is reduced.

The investigations in [77] also lead to the detection of certain limitations of rectangular pulses when the aim is to achieve a flexible and efficient beamforming with TMA. Such vulnerabilities are directly related to the frequency response of rectangular pulses (Chapter 3, p89, [78]): a minimum main-lobe width and a modest side-lobe level (−13 dB), together with a slow (first order) asymptotic side-lobe decay. In fact, taking as the starting point the weaknesses of rectangular pulses when applied to harmonic beamforming, the following two contributions were introduced in [79]:

- A type of pulses that are more suitable for harmonic beamforming with TMA: the so-called sum-of-weighted-cosine (SWC) pulses [80].
- Based on the previous pulses, a new family of beamforming TMA, termed ETMA, is characterized and evaluated in terms of efficiency by properly comparing it to conventional beamforming TMA based on rectangular pulses.

**Figure 5:**
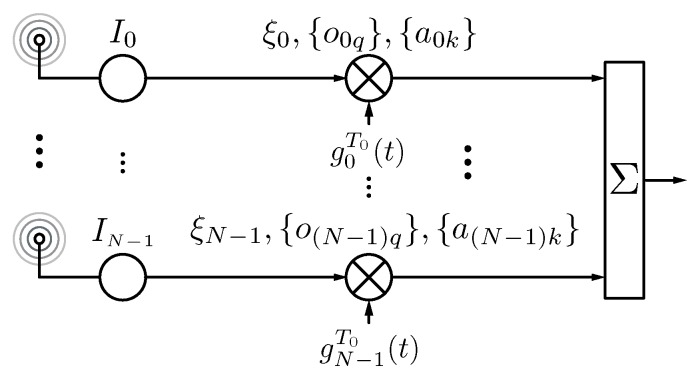
Block diagram of a TMA governed by periodical sum-of-weighted-cosine (SWC) pulses $g_{n}^{T_{0}}\left(t\right)$.

Figure 5 shows the block diagram of an ETMA where the static excitations $I_{0},\cdots ,I_{N-1}$ of the array are time-modulated by more sophisticated periodical waveforms parametrized by $\xi_{n}$ (normalized pulse durations), $o_{nq}$ (normalized pulse time shifts), and $a_{nk}$ (pulse weights), n∈{0,1,⋯,N−1}, q∈{0,±1,⋯,±L}, and k∈{0,1,⋯,K}, where *N* is the number of antenna elements, *L* is the order of the highest exploited harmonic, and *K* is the order of the pulse. A common denominator of these recent investigations [72,75,77,79] is that TMA are analyzed and evaluated not only from an antenna outlook, but also from a signal processing perspective.

## 3. Challenges and Future Research Lines

The interplay between TMA and digital communications still faces a number of challenges. Among them, we highlight the following ones:

- The exploitation of TMA at transmission. Up to now, the applications of TMA in the area of digital communications mainly focus on receiving TMA. Hence, the performance of transmitting TMA from a signal processing outlook, and in different scenarios, is still an unexplored research field. We propose two areas which certainly deserve further exploration: (1) the performance analysis of transmitting TMA in multiuser scenarios; and (2) the feasibility of diversity transmission techniques with TMA.
- Performance with broadband signals. The TMA state of the art exclusively focuses on narrowband signals. However, communications nowadays must unavoidably deal with broadband signals. The higher the bandwidth, the higher the switching frequency in the TMA, the wider the bandwidth at the RF stage, and the higher the sampling rate at the ADC. On the other hand, an analysis of TMA behavior under frequency-selective fading still remains to be done.
- Beamforming design through the preprocessing of periodic pulses. More specifically, we propose a more accurate design of the periodic pulses in the frequency domain. The Fourier transform of a periodic pulse is a discrete spectrum with impulses at multiples of the time-modulation frequency and whose corresponding areas are 2π times the associated exponential Fourier series coefficients. Therefore, a simpler design, by applying the Fourier series coefficients properties to preprocess conventional rectangular pulses before they are applied to the antenna elements, is possible.

## 4. Conclusions

The state of the art of TMA is organized in this work from two perspectives depending on the antenna synthesis criteria: (1) designs from an antenna perspective (exclusively) or (2) designs from a signal processing perspective. Most of the research works about TMA belong to the first group and are characterized by studying the TMA in an isolated manner, or, in other words, exclusively under the antenna designer point of view. The second group consists of those investigations that consider, in addition, the interaction between the TMA technique and the nature of the signals sent or received by such an antenna, i.e., those works with a strong component of array signal processing and, therefore, constituting the hybrid discipline antennas-signal processing. The most recent works belong to the latter group and definitely are marking the future lines of investigation in this promising topic.

## Abbreviations

ADC	analog-to-digital converter
AM	amplitude modulation
AWGN	additive white Gaussian noise
BER	bit error rate
BPSK	binary phase shift-keying
DAC	digital-to-analog converter
DOA	direction of arrival
ETMA	enhanced time-modulated array
FM	frequency modulation
LBFN	linear beamforming network
LFM	linearly frequency modulated
MRC	maximum ratio combining
RF	radio frequency
SLL	side-lobe level
SNR	signal-to-noise ratio
SPDT	single-pole double-throw
SR	sideband radiation
SWC	sum-of-weighted-cosine
TMA	time-modulated array
TMRA	time-modulated reflector array
